# Circulating TMAO, the gut microbiome and cardiometabolic disease risk: an exploration in key precursor disorders

**DOI:** 10.1186/s13098-024-01368-y

**Published:** 2024-06-17

**Authors:** Saba Naghipour, Amanda J. Cox, Joshua J. Fisher, Manuel Plan, Terra Stark, Nic West, Jason N. Peart, John P. Headrick, Eugene F. Du Toit

**Affiliations:** 1 School of Pharmacy and Medical Sciences, Griffith University, Southport, QLD 4215 Australia; 2 Menzies Health Institute Queensland, Griffith University, Parklands Drive, Southport, QLD 4215 Australia; 3 School of Medicine and Public Health, The University of Newcastle, Callaghan, NSW 2308 Australia; 4 Metabolomics Australia (Queensland Node), The University of Queensland, St. Lucia, QLD 4072 Australia; 5 Metabolomics Facility, QIMR Berghofer Medical Research Institute, 300 Herston Rd, Herston, QLD 4006 Australia

**Keywords:** Cardiometabolic disease, Diet, Gut microbiota, Metabolic syndrome, Obesity, Trimethylamine-N-oxide, Diabetes

## Abstract

**Background:**

Elevations in the gut metabolite trimethylamine-N-oxide (TMAO) have been linked to cardiovascular and metabolic diseases. Whether elevated TMAO levels reflect early mechanistic involvement or a sequela of evolving disease awaits elucidation. The purpose of this study was to further explore these potential associations.

**Methods:**

We investigated relationships between circulating levels of TMAO and its pre-cursor substrates, dietary factors, gut microbiome profiles and disease risk in individuals with a Healthy BMI (18.5 < BMI < 25, *n* = 41) or key precursor states for cardiometabolic disease: Overweight (25 < BMI < 30 kg/m^2^, *n* = 33), Obese (BMI > 30, *n* = 27) and Metabolic Syndrome (MetS; ≥ 3 ATPIII report criteria, *n* = 39).

**Results:**

Unexpectedly, plasma [TMAO] did not vary substantially between groups (means of 3–4 µM; *p* > 0.05), although carnitine was elevated in participants with MetS. Gut microbial diversity and *Firmicutes* were also significantly reduced in the MetS group (*p* < 0.05). Exploratory analysis across diverse parameters reveals significant correlations between circulating [TMAO] and seafood intake (*p* = 0.007), gut microbial diversity (*p* = 0.017–0.048), and plasma [trimethylamine] (TMA; *p* = 0.001). No associations were evident with anthropometric parameters or cardiometabolic disease risk. Most variance in [TMAO] within and between groups remained unexplained.

**Conclusions:**

Data indicate that circulating [TMAO] may be significantly linked to seafood intake, levels of TMA substrate and gut microbial diversity across healthy and early disease phenotypes. However, mean concentrations remain < 5 µM, with little evidence of links between TMAO and cardiometabolic disease risk. These observations suggest circulating TMAO may not participate mechanistically in cardiometabolic disease development, with later elevations likely a detrimental sequela of extant disease.

**Supplementary Information:**

The online version contains supplementary material available at 10.1186/s13098-024-01368-y.

## Introduction

A growing body of evidence implicates gut bacteria in the linkages between diet and cardiovascular [1] and other chronic diseases [2]. Dietary composition strongly influences the gut microbiome profile, owing to varying micro- and macro-nutrient requirements of different bacteria [3]. Resultant shifts in the gut biome may, in turn, promote cardiometabolic disease development [4]. Recently the microbial metabolite TMAO has garnered attention as a mediator of this gut-chronic disease connection, following evidence of significant elevations in cardiovascular disease (CVD) [1]. Subsequent studies report associations between TMAO and cardiometabolic and renal disorders, including atherosclerosis [1], obesity [5], type 2 diabetes mellitus (T2DM) [6], heart failure [7] and chronic kidney disease [8]. Whether such associations reflect causal involvement in initial disease development, or a role for TMAO as a disease biomarker, remains unclear [9]. Circulating [TMAO] in healthy humans ranges from 2 to 5 μM [1, 3, 10–12], and although modest elevations up to ~ 10 µM are reported in obesity [5], T2DM [6], heart failure [13] or advanced age [14], these concentrations appear to fall below pathological thresholds in human and animal cells [9]. Indeed, as we have recently reviewed [9], human studies linking TMAO to CVD implicate pathogenic influences at or below concentrations observed in healthy cohorts, whereas the high [TMAO] shown to induce pathological effects experimentally are only achieved in end-stage renal failure. Other investigations fail to link [TMAO] and coronary disease risk in 33–55 year olds without CVD [15], and find no associations between [TMAO], infarct history, coronary disease or major adverse cardiovascular events over an 8 year follow-up period in those with suspected CVD [16].

Contributing to uncertainties regarding possible roles of TMAO, regulation of its formation and circulating concentrations are poorly understood, in both health and disease. Apart from direct absorption from seafood [12, 17], TMAO is largely formed via microbial metabolism of choline [12] and carnitine [3], primarily of animal origin, and subsequent *N*-oxidation of generated TMA by hepatic flavin-containing monooxygenase 3 (FMO3) [1]. Betaine is an additional source, chiefly from betaine-rich foods such as wheat brans/germs, fungi and spinach. The makeup of the gut microbiome, coupled with dietary patterns, are thus important in determining TMAO generation [18]. For example, bacterial taxa including *Prevotella*, *Deferribacteres* and *Teneriticutes* species can metabolise choline and carnitine to TMA [3, 19], and people with a *Prevotella* enriched enterotype may generate higher levels of TMAO than those with a *Bacteroides* enriched enterotype [3]. Gene analysis indicates TMA production may be favoured in *Firmicutes*, *Proteobacteria* and *Actinobacteria* species, but not *Bacteroidetes* [20, 21]. Nonetheless, the interactions between diet and gut bacteria in governing TMAO generation remain to be detailed [9]. That said, plant-based diets (thus lower TMAO substrate intake) are linked to distinct gut microbiota profiles and lower circulating [TMAO] [3, 22]. Other work indicates red *vs*. white meat may specifically increase bacterial metabolism of carnitine to TMA, and also reduce TMAO excretion [23]. Conflicting findings nonetheless exist: for example, neither choline supplementation nor egg consumption influences circulating [TMAO] or gut bacterial diversity in patients with MetS [24]. Moreover, while seafood is the strongest dietary determinant of circulating [TMAO], routine consumption is linked to reduced rather than increased CVD risk [25].

This study explores relationships between circulating [TMAO], its substrate concentrations, dietary factors, gut microbiome diversity, and cardiometabolic disease risk across major precursor states for both CVD and T2DM, including overweight, obesity and MetS. We reasoned that a focus on these underpinning disorders better allows a test of TMAO's potential role in disease development, avoiding the complicating influences of extant and worsening disease (e.g. via renal, hepatic and other dysfunctions) on TMAO generation/handling. Our results indicate that circulating [TMAO] remains below pathological thresholds in these metabolic disorders, with no significant links between [TMAO] and cardiometabolic disease risk. This suggests that significant elevations in [TMAO] in existing disease may reflect a disease sequela (which may in turn promote dysfunction) rather than early pathogenic mechanism.

## Methods

### Human ethics and participants

A retrospective analysis was conducted in plasma samples (collected between 06:00 and 10:00 following an overnight fast) obtained over a 5-year period by the Mucosal Immunology Research Group (Griffith University) as part of a series of studies exploring immune, inflammation and metabolic signalling in health and disease [26, 27]. Parent studies were conducted with approval from the Griffith University Human Research Ethics Committee (ref# 2013/868, 2014/537, 2015/229, 2017/646) and in accordance with the Declaration of Helsinki. Concentrations of TMAO and precursor substrates were determined in plasma samples, with potential relationships between patient physical and biochemical measures, dietary makeup and gut microbiome composition explored.

**Fig. 1:**
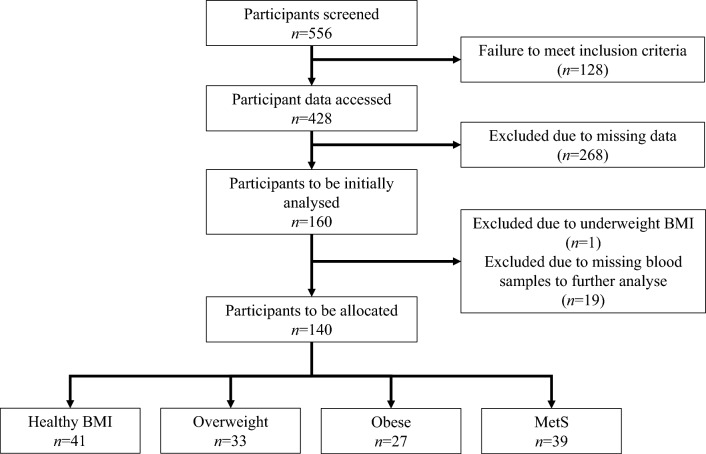
Consort diagram of participant recruitment and grouping. Participant data was originally accessed and screened with participants excluded based on missing data or samples. The remaining participants (*n* = 140) were then accordingly grouped

Participants were recruited from the general population: inclusion criteria included participants aged between 18 and 76 years; exclusion criteria included pregnancy, current infectious illness, history of liver or kidney disease, use of immunomodulating medications, current consumption of probiotic supplements, or being underweight (BMI < 18.5). Participants were initially screened (*n* = 556) and excluded (*n* = 128) according to these criteria. Remaining participant data was accessed (*n* = 428) and individuals further excluded due to missing data (*n* = 268). From the remaining participants (*n* = 160), further exclusions were made based on underweight BMI (*n* = 1) or missing blood samples (*n* = 19). The final 140 participants were divided into 4 groups: (i) Healthy BMI (18.5 < BMI < 25 kg/m^2^ *n* = 41); (ii) Overweight (25 < BMI < 30 *n* = 33); (iii) Obese (BMI > 30 kg/m^2^ *n* = 27); (iv) and MetS (*n* = 39). The latter MetS was classified based on criteria established in the National Cholesterol Education Program Adult Treatment Panel III report (ATPIII), which includes the presence of ≥ 3 of: elevated triglycerides (≥ 1.7 mmol/L) or drug treatment for hypertriglyceridemia; reduced HDL cholesterol (< 1.04 mmol/L for men or < 1.30 mmol/L for women) or treatment for low HDL; elevated BP (systolic ≥ 130 or diastolic ≥ 85 mmHg) or treatment with anti-hypertensives; fasting hyperglycemia (plasma glucose ≥ 5.6 mmol/L); and high waist circumference (> 102 cm for men, > 88 cm for women) [28]. A consort diagram is provided, detailing the flow of participant recruitment, screening and grouping (Fig. 1).

### Participant characterisation

Anthropometric and physiological data included: height, to the nearest half centimetre using a wall-mounted stadiometer (Surgical and Medical Products, NSW, Australia); body mass, using a digital body composition scale (modelHBF-202, Omron Australia, Melbourne, Australia); waist and hip circumference, assessed in accordance with the World Health Organisation Stepwise approach using a graduated anthropometric measuring tape (Seca, Germany); and BP and pulse rate, determined using an automatic BP monitor (model HEM-7121, Omron Australia, Melbourne, Australia) in seated individuals. Blood pressure was further graded according to American College of Cardiology and American Heart Association guidelines [29]: normal (systolic BP < 120 mmHg, diastolic BP < 80 mmHg); elevated (systolic BP 120–129 mmHg, diastolic BP < 80 mmHg); Stage 1 hypertension (systolic BP 130–139 mmHg or diastolic BP 80–89 mmHg); and Stage 2 hypertension (systolic BP ≥ 140 mmHg and diastolic BP ≥ 90 mmHg). The Australian Type 2 Diabetes Risk Assessment Tool (AUSDRISK) was also used to calculate risk of T2DM development based off a self-reported questionnaire. Participants are allocated a score based off their responses to the questions which assess age, sex, ethnicity, family history of T2DM, hyperglycaemia, medication prescriptions, smoking status, exercise habits, and waist measurements. Participants are then grouped into either a “low risk”, “intermediate risk”, or “high risk” based off their scores. Macro- and micronutrient intake was recorded from 3-day self-reported food diaries and analysed using FoodWorks Software (Xyris, Brisbane, Australia), with daily average intakes estimated.

### Blood biomarker assessment

Samples were analysed within 12 h of collection, including full blood count, white cell differential and glycated haemoglobin (HbA1c). Analysis was outsourced to a local pathology provider (QML Pathology, Murarrie, Queensland, Australia). Serum samples were analysed for cholesterol, triglycerides, HDL cholesterol, glucose, and C-reactive protein (CRP) on a COBAS Integra 400 system using commercially available reagents, calibrators and controls (Roche Diagnostics, NSW, Australia). Low density lipoprotein (LDL) cholesterol was determined using the Friedewald equation [30]. Insulin concentrations were measured using a Diabetes 10-plex multiplex assay and Bioplex suspension array system (Bio-rad Laboratories, California, USA).

### Gut microbial composition

Participant faecal samples were used for microbial profiling using universal primers for the V3–V4 region of microbial 16 s rRNA (341F: 5′-CCTACGGGNGGCWGCAG-3′; 805R: 5′GACTACHVGGGTATCTAATCC-3′), as detailed previously [31]. The PCR products were sequenced on an Illumina MiSeq system (Illumina, CA, USA) by a commercial provider, with data processed according to their established in-house data processing pipelines (Macrogen, Seoul, Korea). Briefly, sequence data were processed with CD-HIT-OTU [32] to filter out erroneous and chimeric reads. Taxonomic classification and identity assignment was performed using a reference-based approach with the NCBI BLAST database of 16 s rRNA gene sequences. Gut microbial richness and diversity were considered using operational taxonomic units (OTU), and α-diversity metrics: Chao1, Shannon and inverse Simpson.

### Liquid chromatography-tandem mass spectrometry (LC–MS/MS) analysis

#### Sample preparation

Blood plasma samples were thawed on ice and kept cool at all times. A 200 µL sample was filtered through a 3 kD cut-off spin membrane (Amicon Ultra 0.5 mL, UFC5003BK) at 14,000 g for 30 min at 4 °C. Internal standard (1 µL of 500 µM azidothymidine) was added to 100 µL of filtered sample or standard mix in a HPLC glass insert. The samples within replicates were fully randomised for analysis to reduce batch effect-based bias, forming completely randomised block design analytical experiments. Samples were flanked with repeat injections of standards and pooled QC samples to monitor instrument stability and ensure data integrity [33].

#### Instrumentation and metabolite analysis

Targeted LC–MS/MS metabolomics analyses were performed using a Shimadzu ultra-high performance liquid chromatography (UHPLC) system coupled to a Shimadzu 8060 triple quadrupole mass spectrometer. The UHPLC (Nexera X2, Shimadzu Corp., Kyoto, Japan) consisted of LC-30AD pump units, DGU-20ASR degassing units, a SIL-30AC autosampler, a CTO-20AC column oven, a CBM-20A communications BUS module and an FCV-20AH2 diverter valve unit.

Liquid chromatography was performed using a Shim-pack Velox SP-C18 UHPLC column (2.7 um, 2.1 × 150 mm, PN: 227-32009-04, Shimadzu) with a guard column (SecurityGuard Gemini-NX C18, 4 × 2 mm, PN: AJO-8367, Phenomenex). Solvent A and B were 0.1% formic acid in water or acetonitrile (Lichrosolv, PN: 1142914000, Merck), respectively. Chromatography was performed at 300 µL/min flow-rate using the gradient detailed in Supplementary Table 1. Samples were kept at 4 ℃ in the autosampler and the column was operated at 40 ℃ in the column oven. Sample volumes analysed were 5 and 10 µL.

The Shimadzu 8060 QqQ system had an electrospray ion source, and used N_2_ (> 99.999 vol % BOC Australia, North Ryde, NSW 2113) and Ar (> 99.999 vol %, UN1006, Coregas Pty Ltd, Yennora NSW 2161) as drying and collision gases, respectively. Further instrument details include: drying gas flow, 10 L/min; nebulising gas flow, 3.0 L/min; heating gas flow, 10 L/min; desolvation line, 250 ℃; heat block temperature, 400 ℃; CID gas, 270 kPa; interface temperature, 300 ℃. Interface potential was optimised by performing scheduled multiple reaction monitoring experiments on the standard mix at 0.5, 0.75, 1.0, 2.0, 3.0 kV to determine peak response and obtain lowest limits of detection (LOD) possible. Data were collected from 0 to 3.5 min and flow diverted to waste from 5 to 25 min during column clean up and re-equilibration.

Scheduled multiple reaction monitoring transitions were optimised on positive ionisation mode (m/z + H) for 7 compounds including: TMAO, fully deuterium-labelled TMAO (TMAO-D9), TMA, betaine, carnitine, choline, and azidothymidine. Analytical standards were sourced from Sigma-Aldrich. Details of compound and instrument parameters are in Supplementary Table 2.

### Data and statistical analyses

All data were analysed using GraphPad Prism 9 and are presented as mean ± SEM. Grubbs’ test was performed to identify outliers in the data for removal. Normality of data was checked by a Shapiro–Wilk test. To test for potential associations with [TMAO], a Pearson’s correlation was performed with all variables against TMAO concentrations in initial exploratory analysis.

Student’s t-test was used to determine specific differences between 2 groups, while 1- or 2-way analysis of variance (ANOVA) was used when comparing more than 2 groups (where participants were appropriately categorised into their study groups). A Dunnet’s post-hoc test was employed when comparing data to the Healthy BMI group; a Šidák’s post-hoc for 2-ANOVA comparisons across groups; and a Tukey’s post-hoc test was used for all comparisons. A Cramér's V was also conducted with a corresponding 95% confidence interval to measure association between 2 nominal variables for T2DM risk. A *p*-value of < 0.05 was indicative of statistical significance across tests. Values of *p* < 0.10 are highlighted as potentially relevant biological responses worthy of study, in accordance with recommendations of the American Statistical Association [34]. To highlight the drawbacks to unnecessary correction for multiple comparisons in a wide-ranging exploratory analysis with large numbers of end-points [35–37], we additionally provide q-values, determined via the Benjamini, Krieger and Yekutieli method [38], for the correlations in Table 3.

## Results

### Participant characteristics and T2DM risk

Anthropometry, blood biochemistry and physiological data for 140 participants are summarised in Table 1, segregated into 4 groups: Healthy BMI (*n* = 41), Overweight (*n* = 33), Obese (*n* = 27) and MetS (*n* = 39). For the purposes of this study the 'Healthy' group was based specifically on BMI criteria, and individuals within the group may exhibit disease risk factors. In particular, approximately half appeared pre- or hypertensive, though none exhibited abnormal glucose levels. Three participants from the Healthy BMI group exhibited plasma LDL > 4.5 mmol/l, and two a CRP level of 10 mg/L (suggesting a pro-inflammatory state [39]). Participants with MetS were older (~ 5 yrs) when compared against those with obesity, and individuals with either obesity or MetS generally exhibited a combination of known risk markers for metabolic disease (Table 1).

**Table 1:** Study participant characterisation: anthropometry, blood biochemistry, lifestyle and disease history

	Healthy BMI	Overweight	Obese	MetS	*p values*
Anthropometric measures					
Number (M/F)	41 (18/23)	33 (14/19)	27 (11/16)	39 (29/10)	
Age (yr)	47.8 ± 1.9	44.0 ± 1.9	45.7 ± 1.7	51.9 ± 1.9*	*, *p* < 0.05 *vs*. Obese
Aboriginal, Torres Strait Islander, Pacific Islander, or Maori descent (total participant)	0	0	1	2	
Body weight (kg)	67.7 ± 1.4	78.2 ± 1.7**	101.1 ± 2.6^$,#^	99.7 ± 2.8^$,#^	**, *p* < 0.005 *vs*. Healthy BMI; $ *p* < 0.0001 *vs.* Healthy BMI; #, *p* < 0.0001 *vs.* Overweight
MetS criteria (/5)	0.39 ± 0.11	0.82 ± 0.13	1.89 ± 0.08	3.51 ± 0.11	
BMI (kg/m^2^)	22.7 ± 0.2	26.8 ± 0.2^$^	34.1 ± 0.7^$,#^	32.9 ± 0.7^$,#^	$, *p* < 0.0001 *vs.* Healthy BMI; #, *p* < 0.0001 *vs.* Overweight
Waist (cm)	79.4 ± 1.0	87.4 ± 1.3**	105.4 ± 2.1^$,#^	110.4 ± 2.1^$,#^	**, *p* < 0.005 *vs.* Healthy BMI; $, *p* < 0.0001 *vs.* Healthy BMI; #, *p* < 0.0001 *vs.* Overweight,
Systolic BP (mm/Hg)	118.5 ± 2.4	126.0 ± 1.5	134.0 ± 2.3**	137.1 ± 2.2^$.^*	*, *p* < 0.05 *vs*. Overweight; **, *p* < 0.005 *vs.* Healthy BMI; $, *p* < 0.0001 *vs.* Healthy BMI
Diastolic BP (mm/Hg)	76.7 ± 1.2	82.4 ± 1.9*	89.7 ± 1.3**^,$^	91.0 ± 1.5^+,$^	*, *p* < 0.05 *vs*. Healthy BMI; **, *p* < 0.01 *vs*. Overweight; + , *p* < 0.05 *vs.* Overweight; $, *p* < 0.0001 *vs.* Healthy BMI
BP category (%)					
Normal	48.8	24.2	0	2.6	
Elevated	17.1	15.2	3.7	2.6	
Stage 1	29.7	45.5	59.3	56.4	
Stage 2	4.8	15.2	37.0	38.5	
HbA1c Fraction (%)	5.13 ± 0.05	5.16 ± 0.04	5.21 ± 0.06	5.36 ± 0.05*, **	*, *p* < 0.05 *vs*. Overweight; **, *p* < 0.005 *vs*. Healthy BMI
Glucose (mmol/L)	4.6 ± 0.1	4.8 ± 0.1	5.1 ± 0.1**^,$^	5.5 ± 0.1^+,$^	**, *p* < 0.005 *vs.* Healthy BMI; + , *p* < 0.01 *vs*. Obese; $, *p* < 0.0001 *vs.* Healthy BMI & Overweight
Insulin	6.1 ± 1.6	5.9 ± 1.1	9.9 ± 2.0	11.8 ± 1.3*	*, *p* < 0.05 *vs.* Healthy BMI & Overweight
Cholesterol (mmol/L)	5.18 ± 0.16	5.42 ± 0.16	5.79 ± 0.21*	5.43 ± 0.18	*, *p* < 0.05 *vs.* Healthy BMI
Triglycerides (mmol/L)	0.79 ± 0.05	1.00 ± 0.09	1.30 ± 0.15*	1.77 ± 0.15*^,$^	*, *p* < 0.05 *vs*. Healthy BMI & Obese; $, *p* < 0.0001 *vs.* Healthy BMI & Overweight
HDL (mmol/L)	1.71 ± 0.07	1.64 ± 0.06	1.44 ± 0.06	1.22 ± 0.04*^,$^	*, *p* < 0.05 *vs.* Obese; $, *p* < 0.0001 *vs.* Healthy BMI, Overweight
LDL (mmol/L)	2.80 ± 0.14	3.00 ± 0.15	3.58 ± 0.18*	3.22 ± 0.21	*, *p* < 0.05 *vs*. Healthy BMI
CRP (mg/L)	1.46 ± 0.42	1.21 ± 0.24	2.78 ± 0.56*	2.19 ± 0.34	*, *p* < 0.05 *vs.* Overweight
Urea (mmol/L)	5.23 ± 0.23	5.49 ± 0.28	5.32 ± 0.22	6.00 ± 0.26	NS
Creatinine (umol/L)	68.1 ± 1.6	72.5 ± 2.2	68.9 ± 2.3	77.3 ± 2.1*,**	*, *p* < 0.05 *vs.* Obese; **, *p* < 0.01 *vs*. Healthy BMI
eGFR (mL/min)	88.1 ± 0.8	87.9 ± 0.8	88.3 ± 0.9	86.5 ± 1.2	NS
ALP (U/L)	53.6 ± 1.8	64.2 ± 3.5	72.7 ± 4.0	69.1 ± 2.6	NS
Gamma GT (U/L)	19.3 ± 1.6	25.2 ± 3.2	25.4 ± 2.0	34.2 ± 3.0*	***, *p* = 0.0001 *vs.* Healthy BMI
ALT (U/L)	21.8 ± 1.2	25.5 ± 2.1	32.3 ± 2.3**	32.5 ± 2.9**	**, *p* < 0.01 *vs*. Healthy BMI
AST (U/L)	24.5 ± 1.2	28.7 ± 1.8	30.2 ± 2.3	27.7 ± 1.8	NS
LDH (U/L)	174.0 ± 5.6	188.1 ± 5.6	181.1 ± 5.7	187.2 ± 3.6	NS
Total Protein (g/L)	70.6 ± 0.8	70.3 ± 0.7	71.0 ± 0.7	70.9 ± 0.6	NS
Albumin (g/L)	42.5 ± 0.4	42.2 ± 0.4	42.7 ± 0.4	44.8 ± 0.6*,**,***	*, *p* < 0.05 *vs.* Obese; **, *p* < 0.005 *vs*. Healthy BMI; ***, *p* < 0.001 *vs*. Overweight
Lifestyle					
Smoking (total participant)	1	1	2	3	
Average alcoholic drink consumption (per week)	6.1 ± 0.7	7.2 ± 1.7	5.1 ± 0.9	5.3 ± 1.0	
Exercise habits (> 2.5 h per week)	34	28	18	29	
Patient History (prior physician diagnosis):					
AMI	0	0	0	3	
IBS (total participant)	5	5	3	3	
MetS (total participant)	0	0	0	0	
T2DM (total participant)	0	0	0	0	
Hypertension (total participant)	2	5	3	4	
Hypercholesterolaemia (total participant)	5	5	5	11	
T2DM Risk					
Low (Total participant)	19	11	1	5	
Medium (Total participant)	17	15	6	9	
High (Total participant)	2	9	25	22	

Anthropometric and blood biochemistry data were acquired on entry into the study. Lifestyle and patient history were acquired via questionnaires. Data is reported as mean ± SEM. P-values shown for different inter-group comparisons

*ALP* alkaline phosphatase, *ALT*, alanine transaminase, *AMI* Acute myocardial infarction, *CRP* C-reactive protein, *eGFR* estimated glomerular filtration rate, *IBS* Irritable bowel syndrome, *Gamma GT* gamma-glutamyl transferase, *HDL* high-density lipoprotein, *LDL* low-density lipoprotein, *MetS* Metabolic syndrome, *NS* not significant, *T2DM* Type 2 diabetes mellitus

Lifestyle characteristics and clinical history, including existing physician-diagnosis of relevant cardiometabolic conditions, (Table 1) were determined from self-reported questionnaires. Smoking was more common in people with MetS, while alcohol consumption was similar across groups. Those with a healthy BMI exercised most. Three participants had a history of acute myocardial infarction (AMI), whilst past irritable bowel syndrome (IBS) diagnosis was similar across groups. No participants had been previously diagnosed with either MetS or T2DM. Diagnosis of hypertension was most prevalent in those who were overweight, with hypercholesterolaemia diagnosis highest in individuals with MetS. Participants with either Obesity or MetS exhibited increased risks of developing T2DM (Table 1). A more detailed analysis is provided in the supplement (Fig. S1). Participant dietary intake and medication details are provided in Supplementary Table 3 and 4, respectively.

### Gut microbiome diversity and composition

**Fig. 2:**
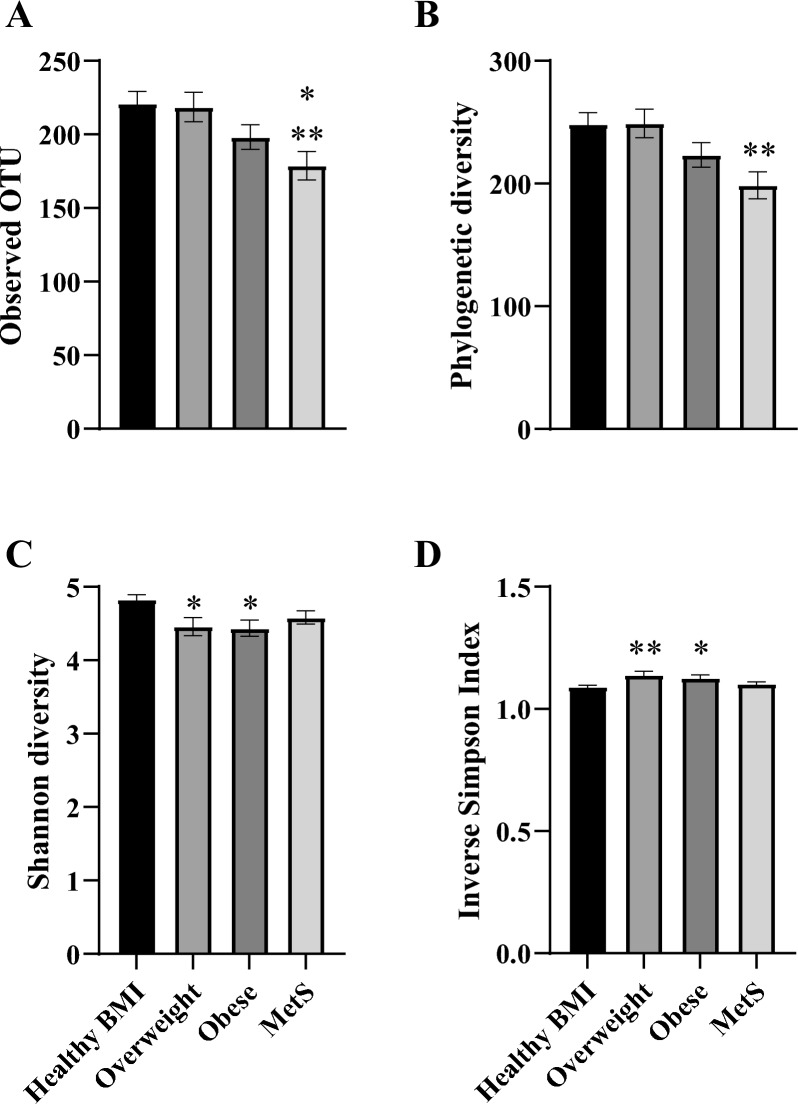
Gut microbiome diversity metrics. **A** Operational taxonomic unit diversity (*, *p* < 0.05 *vs*. Overweight; **, *p* < 0.01 *vs*. Healthy BMI). **B** Chao1 measure of diversity (**, *p* < 0.01 *vs*. Healthy BMI & Overweight). **C** Shannon diversity metric (*, *p* < 0.05 *vs*. Healthy BMI). D) Inverse Simpson Index (*, *p* < 0.05 *vs*. Healthy BMI; **, *p* < 0.01 *vs*. Healthy BMI). Data are presented as mean ± SEM (*n* = 27–41 for all groups) and compared with a one-way ANOVA using an initial Tukey’s post hoc analysis for all comparisons as an exploratory measure to look for changes between all groups. A subsequent one-way ANOVA was also used to compare all means against the Healthy BMI group in conjunction with a Dunnet’s post-hoc test to increase statistical sensitivity

The OTUs for the gut microbial composition (Fig. 2A) were significantly lower in participants with MetS when compared to individuals with a healthy (*p* < 0.01) or overweight (*p* < 0.01) BMI. Phylogenetic diversity using the Chao1 measure (Fig. 2B) was also significantly lower in people with MetS *vs*. those with a healthy or overweight BMI (*p* < 0.01). The Shannon Index appeared lower in groups that were overweight or with obesity (*p* < 0.05), but not with MetS when compared to those with a healthy BMI (Fig. 2C). Likewise the inverse Simpsons Index (Fig. 2D) was not dissimilar between the healthy BMI and MetS groups, however was decreased for overweight (*p* < 0.01) or obese BMI groups (*p* < 0.05).

**Fig. 3:**
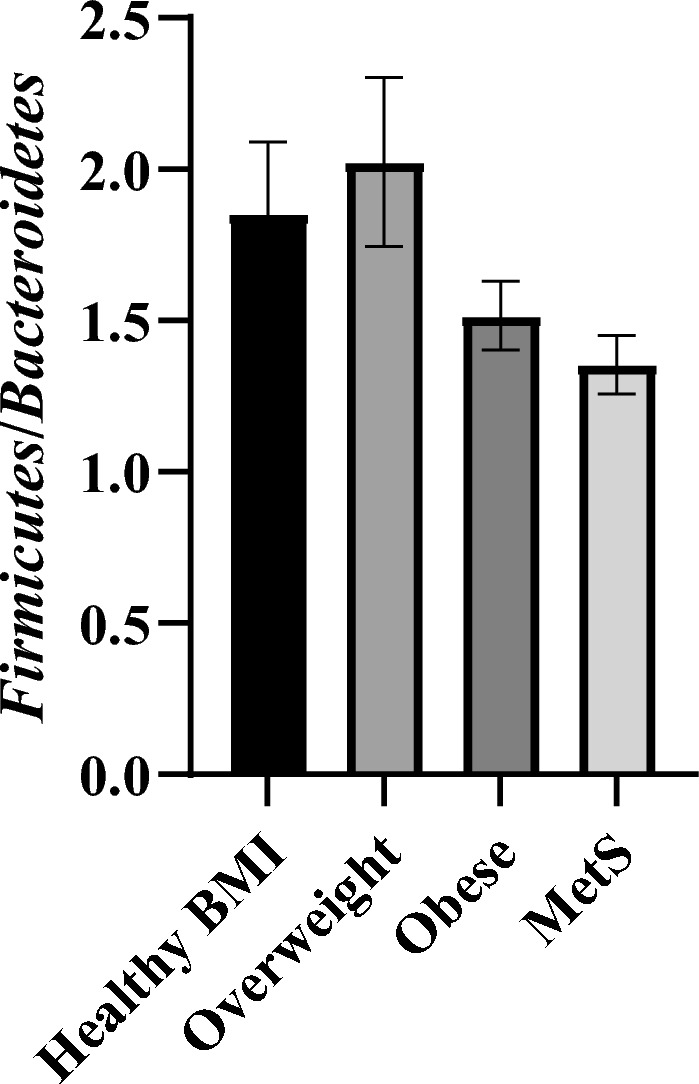
The *Firmicute*/*Bacteroidetes* ratio (FBR). The ratio of *Firmuicutes*:*Bacteroides* bacterial phyla in the gut microbiome was determined for each group. Data are presented as mean ± SEM (*n* = 27–41 for all groups) and compared with a one-way ANOVA using an initial Tukey’s post hoc analysis for all comparisons as an exploratory measure to look for changes between all groups. A subsequent one-way ANOVA was also used to compare all means against the Healthy BMI group in conjunction with a Dunnet’s post-hoc test to increase statistical sensitivity

Gut microbial composition of participants is reported as a relative abundance (Table 2). The two most abundant phyla present were *Bacteroidetes* and *Firmicutes*. Of note, only a handful of participant microbial compositions contained *Cyanobacteria* (< 1%), most of which had a healthy BMI. Individuals with either obesity (*p* < 0.0001) or MetS (*p* < 0.05) contained more *Bacteroidetes* compared with those who exhibited a healthy or overweight BMI. Conversely, only those with MetS exhibited a reduced *Firmicutes* abundance when compared to all other groups (*p* < 0.05–0.0001). A *Firmicutes* to *Bacteroidetes* ratio (FBR) may be used in characterizing the bacterial profile (Fig. 3), and was found to not be different between groups (*p* > 0.05). Similarly, visualisation of overall phyla composition using a partial least squares discriminant analysis (PLS-DA) plot did not reveal marked differences between the groups (Supplementary Fig. 2).

**Table 2:** Gut microbiota phyla composition of participants

Phyla	Healthy BMI	Overweight	Obese	MetS	*p values*
*Actinobacteria*	0.98 ± 0.33%	0.84 ± 0.21%	2.68 ± 1.12%	1.23 ± 0.28%	NS
*Bacteroidetes*	35.16 ± 1.55%	36.03 ± 2.23%	39.32 ± 1.81%*	41.68 ± 1.80%^$^	*, *p* < 0.05 *vs.* Healthy BMI; $ *p* < 0.0001 *vs.* Healthy BMI and Overweight
*Cyanobacteria*	0.00 ± 0.00%	0.00 ± 0.00%	0.00 ± 0.00%	0.00 ± 0.00%	NS
*Firmicutes*	53.96 ± 1.43%	57.13 ± 1.90%	54.64 ± 1.91%	50.41 ± 1.71%*^,$^	*, *p* < 0.05 *vs.* Healthy BMI & Obese; $ *p* < 0.0001 *vs.* Overweight
*Fusobacteria*	0.00 ± 0.00%	0.50 ± 0.50%	0.00 ± 0.00%	1.27 ± 0.92%	NS
*Lentisphaerae*	0.00 ± 0.00%	0.00 ± 0.00%	0.00 ± 0.00%	0.00 ± 0.00%	NS
*Proteobacteria*	4.21 ± 1.08%	1.43 ± 0.29%	1.37 ± 0.37%	3.05 ± 0.67%	NS
*Synergistetes*	0.15 ± 0.11%	0.01 ± 0.01%	0.00 ± 0.00%	0.01 ± 0.00%	NS
*Tenericutes*	0.05 ± 0.03%	0.02 ± 0.02%	0.00 ± 0.00%	0.01 ± 0.00%	NS
*Verrucomicrobia*	5.16 ± 1.15%	3.72 ± 1.18%	1.87 ± 0.69	2.06 ± 0.85%	NS

Bacterial profiles were determined via 16 s rRNA sequencing on faecal content. Data are presented as mean ± SEM and compared with a one-way ANOVA using an initial Tukey’s post hoc analysis for all comparisons as an exploratory measure to look for changes between all groups. A subsequent one-way ANOVA was also used to compare all means against the Healthy BMI group in conjunction with a Dunnet’s post-hoc test to increase statistical sensitivity. P-values shown for different inter-group comparisons

### Circulating TMAO and pre-cursor concentrations

**Fig. 4:**
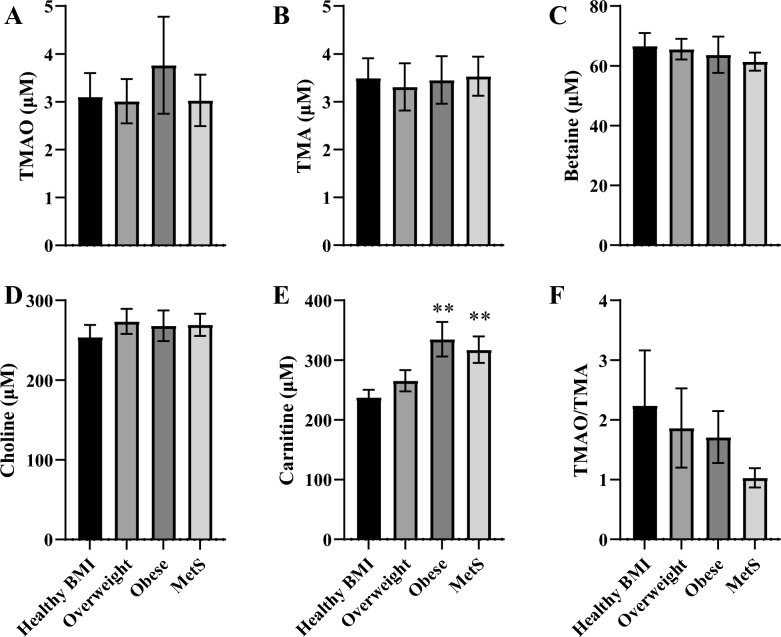
TMAO pathway metabolites in human plasma. TMAO and its precursor substrates measured from fasting plasma. **A** TMAO. **B** TMA. **C** Betaine. **D** Choline. **E** Carnitine. Circulating carnitine was highest in the Obese and MetS group when compared to Healthy BMI participants (*, *p* < 0.01). **F** TMAO: TMA ratio. Data are presented as mean ± SEM (*n* = 27–41 for all groups) and compared with a one-way ANOVA using an initial Tukey’s post hoc analysis for all comparisons as an exploratory measure to look for changes between all groups. A subsequent one-way ANOVA was also used to compare all means against the Healthy BMI group in conjunction with a Dunnet’s post-hoc test to increase statistical sensitivity

Circulating [TMAO] together with precursor substrates (TMA, betaine, choline, and carnitine) were determined under fasting conditions (Fig. 4). The TMAO concentration did not differ significantly between groups (*p* > 0.05), though a trend for slightly higher levels (~ 20%) was evident in those with obesity (Fig. 4A). Similar concentrations of the pre-cursor TMA (Fig. 4B), and substrates betaine (Fig. 4C) and choline (Fig. 4E) were evident between groups, whereas plasma [carnitine] was significantly elevated (*p* < 0.01) in those with obesity and MetS when compared to individuals with a Healthy BMI. The ratio of TMAO to TMA (Fig. 4F) did not differ between groups. Further analysis was conducted to explore potential sex differences (Supplementary Fig. 3), with no differences in [TMAO] detected between males and females.

### Correlation analysis

Broader relationships between all variables were initially explored (Supplementary Fig. 4). However, evidence of clear relationships between most other variables were modest and as such, we chose to feature specific relationships with [TMAO], as the focus of this study. Exploratory correlation analysis was undertaken to test for potential relationships between circulating [TMAO] and a variety of parameters, spanning levels of precursor substrates, together with age, anthropometric and biochemical measures, disease risk and gut microbiome profiles (Table 3). Of the variables explored, circulating [TMAO] was only significantly correlated (*p* < 0.05) with dietary α-tocopherol, iron and intake of seafood (with low long chain N-3 polyunsaturated fatty acid content), gut microbiome diversity, and circulating [TMA]. Correlations at a *p* < 0.10 level were also evident for circulating [choline], markers of liver function (ALP, AST), and dietary energy and polyunsaturated fat intakes. Further work is warranted to test specific hypotheses regarding causal relationships between these parameters. To highlight the drawback to unnecessarily correcting for multiple comparisons in an exploratory analysis such as this [35–37], we additionally present q-values for these associations. Note that this form of correction eliminates the ability of exploratory analysis to identify any potential relationships with [TMAO] (Table 3). It is also notable that no parameter individually explains more than 7% of the variance in TMAO levels (TMA 7%, seafood 5%, iron 5%, bacterial diversity 3–4%), leaving much of the variance in [TMAO] to be explained by other factors. A systems biology or network approach (with multi-regression modelling) is ultimately required to investigate how these (and additional) determinants may interact in an integrated manner to regulate circulating [TMAO].

**Table 3:** Correlations between plasma [TMAO], anthropometric measures, disease risks and microbiome profiles

Parameters	Correlation with circulating [TMAO] (µM)			
	r	r^2^	*p*-value	*q*-value
Age (years)	0.038	0.001	0.657	0.878
*Anthropometric Measures*	0.029	0.001	0.739	0.879
BMI (kg/m^2^)	0.051	0.003	0.555	0.790
Waist (cm)	− 0.005	0.000	0.950	0.962
Systolic BP (mm/Hg)	− 0.055	0.003	0.525	0.786
Diastolic BP (mm/Hg)	− 0.085	0.007	0.318	0.707
*Cardiometabolic Profile*				
HbA1c Fraction (%)	− 0.063	0.004	0.460	0.786
Glucose (mmol/L)	0.008	0.000	0.927	0.962
Insulin	− 0.093	0.009	0.275	0.707
Diabetes risk score	− 0.012	0.000	0.887	0.962
Cholesterol (mmol/L)	− 0.034	0.001	0.691	0.879
Triglycerides (mmol/L)	− 0.088	0.008	0.304	0.707
HDL (mmol/L)	− 0.029	0.001	0.737	0.879
LDL (mmol/L)	− 0.027	0.001	0.750	0.880
CRP (mg/L)	− 0.010	0.000	0.906	0.962
Urea (mmol/L)	0.052	0.003	0.543	0.786
Creatinine (umol/L)	− 0.103	0.011	0.226	0.684
eGFR (mL/min)	0.023	0.001	0.783	0.883
ALP (U/L)	0.159	0.025	**0.063**	0.547
Gamma GT (U/L)	− 0.064	0.004	0.461	0.786
ALT (U/L)	0.071	0.005	0.405	0.758
AST (U/L)	0.144	0.021	**0.092**	0.615
LDH (U/L)	0.005	0.000	0.952	0.962
Total Protein (g/L)	− 0.140	0.019	0.103	0.639
Albumin (g/L)	− 0.083	0.007	0.333	0.707
*Nutritional Parameters*				
Energy (kJ)	0.162	0.026	**0.060**	0.547
Protein (g)	0.091	0.008	0.292	0.707
Total fat (g)	0.133	0.018	0.123	0.673
Saturated fat (g)	0.044	0.002	0.614	0.847
Trans Fatty Acids (g)	0.099	0.010	0.252	0.684
Polyunsaturated fat (g)	0.152	0.022	**0.078**	0.565
Monounsaturated fat (g)	0.162	0.026	0.059	0.547
Cholesterol (mg)	0.054	0.003	0.531	0.786
Carbohydrate (g)	0.060	0.004	0.486	0.786
Sugars (g)	0.090	0.008	0.295	0.707
Starch (g)	0.009	0.000	0.919	0.962
Alcohol (g)	0.133	0.018	0.124	0.673
Dietary fibre (g)	0.123	0.015	0.154	0.684
Thiamine (mg)	0.022	0.001	0.793	0.883
Riboflavin (mg)	0.011	0.000	0.903	0.962
Niacin (mg)	0.008	0.000	0.931	0.962
Vitamin C (mg)	0.103	0.011	0.233	0.684
Vitamin E (mg)	0.110	0.012	0.204	0.684
Tocopherol α (mg)	0.184	0.034	***0.032***	0.463
Vitamin B6 by analysis (mg)	0.054	0.003	0.534	0.786
Vitamin B12 (µg)	0.036	0.001	0.678	0.879
Total folate (µg)	0.082	0.007	0.342	0.707
Folic acid (µg)	0.024	0.001	0.780	0.883
Retinol (µg)	0.100	0.010	0.243	0.684
Beta carotene (µg)	0.048	0.002	0.581	0.814
Sodium (mg)	0.088	0.008	0.306	0.707
Potassium (mg)	0.099	0.010	0.252	0.684
Magnesium (mg)	0.079	0.006	0.363	0.720
Calcium (mg)	0.086	0.007	0.317	0.707
Phosphorus (mg)	0.103	0.011	0.231	0.684
Iron (mg)	0.228	0.052	***0.008***	0.232
Zinc (mg)	0.101	0.010	0.241	0.684
Selenium (µg)	0.082	0.007	0.340	0.707
Iodine (µg)	0.111	0.012	0.199	0.684
Fruit (serve)	0.078	0.006	0.369	0.720
Vegetables (serve)	0.067	0.004	0.448	0.786
Red meats (serve)	0.042	0.001	0.625	0.848
Poultry (serve)	0.029	0.001	0.735	0.879
Eggs (serve)	0.064	0.004	0.462	0.786
Seafood (high LC N3 serve)	0.022	0.000	0.780	0.883
Seafood (low LC N3 serve)	0.231	0.053	***0.007***	0.232
Caffeine (mg)	− 0.006	0.003	0.511	0.786
*Gut Microbial Diversity*				
OTU	0.204	0.042	***0.017***	0.369
Chao1	0.183	0.033	***0.032***	0.463
Shannon	0.170	0.029	***0.048***	0.547
Inverse Simpson	0.117	0.014	0.177	0.684
*Bacterial Phyla*				
*Actinobacteria*	− 0.114	0.013	0.187	0.684
*Bacteroidetes*	− 0.102	0.010	0.234	0.684
*Cyanobacteria*	− 0.033	0.001	0.706	0.879
*Firmicutes*	0.113	0.013	0.189	0.684
*Fusobacteria*	0.077	0.006	0.373	0.720
*Lentisphaerae*	0.109	0.012	0.207	0.684
*Proteobacteria*	0.030	0.001	0.725	0.879
*Synergistetes*	− 0.031	0.001	0.716	0.879
*Tenericutes*	− 0.057	0.003	0.513	0.786
*Verrucomicrobia*	0.055	0.003	0.522	0.786
FBR	0.061	0.004	0.481	0.786
*TMAO Substrates*				
TMA (μM)	0.267	0.072	***0.001***	0.087
Betaine (μM)	− 0.071	0.005	0.410	0.758
Choline (μM)	− 0.155	0.024	**0.070**	0.553
Carnitine (μM)	− 0.116	0.014	0.173	0.684

Data shown for correlation analyses, including: R (Pearsons correlation coefficient) and R^2^, P-values for correlations are shown. To highlight limitations of multiple comparison correction in exploratory analysis of diverse parameters (with no a priori hypotheses), we also provide adjusted Q-values, determined via the Benjamini, Krieger and Yekutieli method [38]. Data analysed using GraphPad Prism 9. A *p*-value in ***italicised bold*** denotes significant correlations at the *p* < 0.05 level (**bold** values denote a trend at the *p* < 0.10 level)

*L-C N3* long chain N-3 polyunsaturated fatty acids

## Discussion

In testing the potential role of TMAO in promoting cardiometabolic disease we assessed relationships between circulating [TMAO] and its substrates, chronic disease risk and phenotypic profiles in major precursor disorders (overweight, obesity and MetS) for CVD and T2DM. Somewhat unexpectedly, mean plasma [TMAO] and concentrations of its substrates were largely comparable across these conditions and remained within normal bounds, although plasma [carnitine] was elevated in those with obesity or MetS. Circulating [TMAO] correlated with seafood intake, dietary iron and α-tocopherol, gut microbial diversity, and plasma [TMA], together with a trend for a correlation with circulating [choline]. However, no associations were evident between TMAO and cardiometabolic disease risk, given that there were no substantial changes in [TMAO] between these metabolic disorder groups, nor a significant correlation between traditional CVD markers and [TMAO]. Considering the critical roles of obesity and MetS in CVD and T2DM, these findings appear inconsistent with a causal role for TMAO in the genesis of cardiometabolic disease, though do not exclude a detrimental role for markedly elevated TMAO in advanced disease.

### Pro-disease phenotypes in groups that are overweight, with obesity and with MetS

A range of known CVD risk factors were assessed here. Observations included: higher prevalence of hypertension in groups with obesity and MetS, in agreement with well-established links between obesity and blood pressure dysregulation [40]; higher prevalence of dyslipidaemia in individuals with MetS, consistent with criteria of elevated triglycerides and decreased HDL cholesterol; and importantly, no differences in markers of renal function (urea, eGFR), with the exception of a modest elevation in plasma creatinine in MetS, broadly supporting maintained renal function across groups. This discounts the potentially strong influences of renal (dys)function on TMAO excretion and concentrations.

### Gut microbiome diversity declines with obesity and MetS

Overall, a pattern of lower microbial diversity was noted when comparing those with MetS against individuals with a healthy or overweight BMI (using OTU and Chao1 metrics), and participants with an overweight and obese BMI (using Shannon and Simpson indices) relative to individuals with a healthy BMI. Differences in the proportion of *Bacteroidetes* and *Firmicutes* in people with obesity or MetS were noted, although no consistent change in the FBR was detected. A diverse microbiome is generally linked to healthy phenotypes [41], and an increase in FBR is a mooted hallmark of obesity [42], though studies also report no change [43] or a decline [44] in this ratio with obesity. *Firmicutes* are more effective than *Bacteroidetes* at extracting energy from food intake, favouring caloric excess and obesogenesis [45]. Data here indicate a decrease in *Firmicutes* and increase in *Bacteroidetes* with obesity and MetS, in agreement with prior analysis in obesity [44]. However, the noted high variability in microbial abundance (ranges of 11–95% for *Firmicutes*, and 1–87% for *Bacteroidetes*) presents a major challenge in identifying relevant and consistent shifts in microbial composition in studies such as this [42], where relationships between downstream microbial-derived and secondary metabolites and disease are explored.

### Limited variance in circulating [TMAO] in metabolic disorders

A fasting plasma [TMAO] of 3–4 μM agrees well with prior measures of 2–5 μM in healthy people [10], and did not vary substantially across the metabolic disorders studied. Elevations beyond this concentration range have been reported in different disease states, though the basis of such elevations is unknown [9]. In addition to dietary makeup [11] and gut microbial composition [3], circulating [TMAO] is influenced by hepatic FMO3 expression [1] and renal function [8], among other factors. However, variance in circulating [TMAO] is only partially explained by these determinants [46], implicating important roles for as yet unidentified mechanisms. This is reflected in the present findings, with the influences of microbial diversity, circulating levels of TMA and its precursors unable to explain most of the variance in [TMAO].

Although there is evidence of associations between TMAO and adipose dysfunction [47], we detect no major differences in [TMAO] between groups with widely varying BMI. This stability of [TMAO] across participants with a healthy or obese BMI contrasts studies in animal models of diet-induced obesity, which report circulating [TMAO] as high as 20 μM [48, 49]. Relevance to human obesity remains to be established, however, given twofold higher baseline [TMAO] in rodents compared to humans [9], and extremely high saturated fat and caloric intakes in these models which are not representative of the human diet. Our observations are also at odds with outcomes from a meta-analysis implicating a positive association between [TMAO] and risk of obesity (indicated by BMI) [50], and a reported relation between degree of obesity and increasing [TMAO] [47].

Despite individual elements of MetS being linked with circulating [TMAO], there is relatively little information available regarding relationships between TMAO and MetS. Here a circulating [TMAO] of ~ 3 μM was observed in those with MetS, not differing substantially from other groups. Prior work suggests that [TMAO] is elevated in patients with MetS, however concentrations nonetheless remained relatively low (2 µM *vs.* 1 μM in those without MetS) [16] and fell well within ranges reported in healthy populations [9, 10] (and below that observed here).

Circulating [carnitine] was elevated in individuals with Obesity or MetS, which might favour increased TMAO generation. Similar to our observations, others report increased levels of carnitine and an insignificant trend for increased [TMAO] in patients with nascent MetS [51]. Whether this elevation in carnitine reflects increased intake and/or altered transport and metabolism is unclear, requiring detailed analysis of intakes and excretion. Importantly, estimates of dietary intake (based on reported red meat intake, a strong source of carnitine [3]) did not differ between people with obesity or MetS (Supplementary Table 3). Whether shifts in carnitine transport and gut metabolism may thus contribute to elevated circulating levels, with saturation of small intestine uptake [3, 17] influencing carnitine delivery to distal sites of microbial metabolism [3], awaits further study.

### Factors significantly linked to circulating [TMAO]

The question of what biological factors are linked to or determine circulating [TMAO] under different conditions remains. We thus undertook an exploratory correlation analysis across a diversity of potentially relevant parameters, with no a priori hypotheses. As such, multiple test correction is not required in the search for potential relationships [35–37]. This analysis indicates circulating [TMAO] is not strongly related to age, anthropometric measures, MetS score, T2DM risk or overall cardiometabolic risk profiles. This is not dissimilar to a prior report that TMAO concentrations are not significantly linked to MetS in adults or children [52]. However, the current findings contrast earlier reports indicating that [TMAO] is significantly associated with anthropometric and metabolic profile markers (though not age) [47, 53]. It has also been suggested that TMAO is associated with an unfavourable MetS profile, specifically in subjects with hyperglycaemia [54]. While further inquiry into the current data might be achieved to correlate [TMAO] with similar parameters within study groups, lack of statistical power would render interpretation inappropriate. Nonetheless, we find no evidence of an association between TMAO and MetS profile; given little evidence of CVD or major metabolic disruption in the MetS group, the lack of a relationship between [TMAO] and MetS (and the low levels detected) suggests TMAO may not be causal in the early development of CVD.

Trends for positive associations (*p* < 0.01) were evident between [TMAO] and liver enzymes (ALP and AST) in the unadjusted analyses, suggesting a link between hepatic dysfunction and TMAO generation (potentially via hepatic FMO3 activity). Others confirm a positive albeit weak association between TMAO and AST [55], and TMAO is also elevated in non-alcoholic steatohepatitis patients [55]. Indeed, TMAO itself can up-regulate FMO3 expression [56], suggesting a positive feedback loop within disease states [9]. Evidence that FMO3 expression is increased by hepatic damage indicates a localised feedback loop which may explain positive associations with liver enzymes observed here.

Most dietary macro- and micronutrients were not linked to [TMAO], although there was evidence of positive associations in the unadjusted analyses with seafood, α-tocopherol and iron intakes, and trends for associations with energy and polyunsaturated fat intake (*p* < 0.10). Total energy intake and circulating [TMAO] have been linked previously [47], though only a weak trend was evident here. This may reflect in part the relatively small differences in both circulating TMAO and energy intakes across the 4 groups studied (Supplementary Table 3), noting that modest differences in energy intake align with known limitations of self-reported dietary intakes. Prior studies indicate that seafood has the greatest potential to increase post-prandial [TMAO] [12], though as previously reviewed by Evans et al. [57] this is highly dependent on fish species, with deep-sea fish having the highest TMAO content. The current data suggests intake of seafood low in long-chain N3 fatty acids are positively associated with [TMAO] (Table 3). Examples of this seafood, consumed and reported by study participants, include squid, prawns/shrimp and white flesh fish. In contrast, shallow-living seafood reportedly have low endogenous TMAO and produce lower post-prandial spikes in TMAO when compared to deep-sea fish, though choline and carnitine were not accounted for [58]. While a diet enriched in red (not white) meat may also increase fasting [TMAO] [23], the present data indicate no associations between circulating [TMAO], fruit and vegetable, red meat, poultry or egg intakes.

In agreement with the importance of gut bacteria, [TMAO] initial unadjusted analysis revealed modest associations with gut microbial diversity. Such associations would suggest somewhat paradoxically, that TMAO generation may increase with greater gut microbial diversity, potentially reflecting distinct roles of select species in TMAO generation. Studies in humans show that TMAO “high producers” have increased Shannon and Chao1 diversity measures, with an increase in FBR [59]. Despite these trends in the unadjusted analyses, no significant association was found between [TMAO] and any of the major phyla or FBR in the current study. Recent work also demonstrates a weak albeit significant association between circulating TMAO and microbiome composition in individuals with obesity presenting with MetS features, but not overt T2DM or ischaemic heart disease [60]. Members from the *Firmicutes* phylum were most strongly associated with TMAO, compared to other phyla [60]. Curiously, we find a decreased *Firmicutes* abundance in those with MetS, and no association between this phylum and circulating TMAO within the overall study population*.* One prior study reports that high TMAO producers have gut microbiomes enriched with *Firmicutes vs*. *Bacteroidetes* and a lower overall diversity, whilst low producers have a near equal ratio of the two phyla [12]. Contrasting our findings, a recent study in a Hispanic community in the USA found no association between α-diversity metrics and TMAO [61]. Differences in geography, ethnicity, socioeconomic and lifestyle factors could contribute to this difference, though further work is needed to resolve. Fennema et al*.* [62] provide a comprehensive review and summary detailing bacterial species involved in TMA formation. It is important to note that no study, thus far, has identified microbes within the human microbiome that are specifically responsible for TMA production [63], and that most association studies are confounded by gut transit time.

The potential for positive and negative associations between [TMAO] and circulating TMA and choline, respectively, were also noted in the unadjusted analyses. The former might be predicted given TMA is the immediate substrate for TMAO generation [1]. On the other hand, TMA (thus TMAO) generation is also partly dependent on microbial metabolism of choline [1, 11], which appeared negatively correlated with [TMAO]. Other work indicates that circulating choline does not correlate with TMAO [64, 65]. Although untested, a negative association could also indicate highly efficient net conversion of choline to TMA and TMAO, effectively lowering choline levels as these products increase in the circulation. Given that physiological processes are regulated by multiple interacting factors in an integrated manner, as opposed to the influences of a single mediator, future work would benefit from integrative or biological network based analyses [60].

### Study limitations

There are several constraints to note in the present analysis. Self-reported questionnaires for diet and other risk factors may often under-report intakes. This could contribute to a lack of clear differences in macro- and micronutrient intakes subjects with obese or MetS, despite evident metabolic and phenotypic differences. Given that the study was also conducted in community-dwelling volunteers at a university campus, disease phenotypes may not have been as severe as participants recruited from clinical or other settings. It is also noted that the healthy comparator group (Healthy BMI) was defined solely on BMI in this analysis. As detailed in the Results, some in the Healthy BMI cohort exhibit individual measures outside of normal ranges, including hypertension together with a handful of subjects with elevated CRP or LDL. This highlights the challenges in identifying ‘control’ groups devoid of risk factors in modern populations. While such heterogeneity may cloud differences between sub-groups, any influences of these parameters or risk factors should nonetheless be revealed in multi-regression analysis. The number of final participants recruited were limited for this study, which unfortunately prohibited more detailed multi-regression modelling and mediation analysis. Finally, gut microbial composition was assessed here for major bacterial phyla, and a deeper interrogation may provide additional insight, particularly for regression and correlation analysis, though this would necessarily increase variable numbers and thus influence statistical power.

### Conclusions

In summary, the current study indicates that circulating [TMAO] is not substantially elevated in obesity or MetS beyond levels in those with a healthy BMI. While circulating [TMAO] correlated with gut microbial diversity, seafood, iron and α-tocopherol intakes, circulating [choline] and [TMA], most variance in [TMAO] remains unexplained. Future investigations may address specific hypotheses regarding the roles of these candidate determinants under different metabolic conditions. Importantly, [TMAO] did not appear linked to cardiometabolic disease risk, suggesting that changes in this metabolite may not be mechanistically important in the development of metabolic disorders underpinning CVD and T2DM. We have previously suggested that TMAO may act as a secondary driver of disease in individuals with underlying pathophysiology [9], and recent evidence TMAO exacerbates renal fibrotic injury with co-morbidities such as hypertension or T2DM supports this hypothesis [60]. Nonetheless, contrasting studies in spontaneously-hypertensive-heart-failure rats suggest that TMAO may improve mortality and systemic phenotypes associated with heart failure [66]. Further work is needed to resolve these fundamental questions, however our findings suggest that TMAO elevations may reflect a detrimental disease sequela, potentially participating in later disease progression.

### Supplementary Information

Supplementary Material 1.

## Data Availability

The raw data supporting the conclusions of this article will be made available by the authors, without undue reservation, to any qualified researcher.

## Abbreviations

ALP: Alkaline phosphatase
ALT: Alanine transaminase
AMI: Acute myocardial infarction
ANOVA: Analysis of variance
ATPIII: Adult Treatment Panel-III
AUSDRISK: Australian Type 2 Diabetes Risk Assessment Tool
BMI: Body mass index
BP: Blood Pressure
CRP: C-reactive protein
CVD: Cardiovascular disease
eGFR: Estimated glomerular filtration rate
FBR: Firmicutes to Bacteroidetes ratio
FMO3: Flavin-containing monooxygenase 3
Gamma GT: Gamma-glutamyl transferase
HDL: High-density lipoprotein
IBS: Irritable bowel syndrome
L-C N3: Long-chain N-3 polyunsaturated fatty acids
LDL: Low density lipoprotein
MetS: Metabolic Syndrome
NS: Not significant
OTU: Operational taxonomic units
T2DM: Type 2 diabetes mellitus
TMA: Trimethylamine
TMAO: Trimethylamine-N-oxide
