# Supplementary material for: Circulating TMAO, the gut microbiome and cardiometabolic disease risk: an exploration in key precursor disorders
Source: Diabetol Metab Syndr. 2024 Jun 17;16:133. doi: 10.1186/s13098-024-01368-y (PMC11181661; doi:10.1186/s13098-024-01368-y)
Supplement: Supplementary file 1 — Supplementary Material 1. [file 13098_2024_1368_MOESM1_ESM.docx]

Participants were graded using scoring criteria from AUSDRISK to determine relative risks of developing T2DM (**S Fig 1**). Participants were assigned a score which would then result in being grouped in either low, medium, or high risk of developing T2DM. Half of those with a healthy BMI were placed in the low-risk category to develop T2DM, while 62% of those with obesity and 77% with MetS were at high risk of developing T2DM. Pearson’s chi-squared test (χ^2^_Pearson_ (6) = 65.70) revealed a statistically significant effect at the 0.05 level (*p*<0.01) for participant grouping and T2DM risk. An effect size of 0.47 indicated a moderately strong relationship between the group and risk score using Cramér's V. Only participants with an overweight or obese BMI did not have a statistically significant *p* value for the chi squared test for homogeneity (*p*>0.05), whereas all other groups had significant associations for risk score.


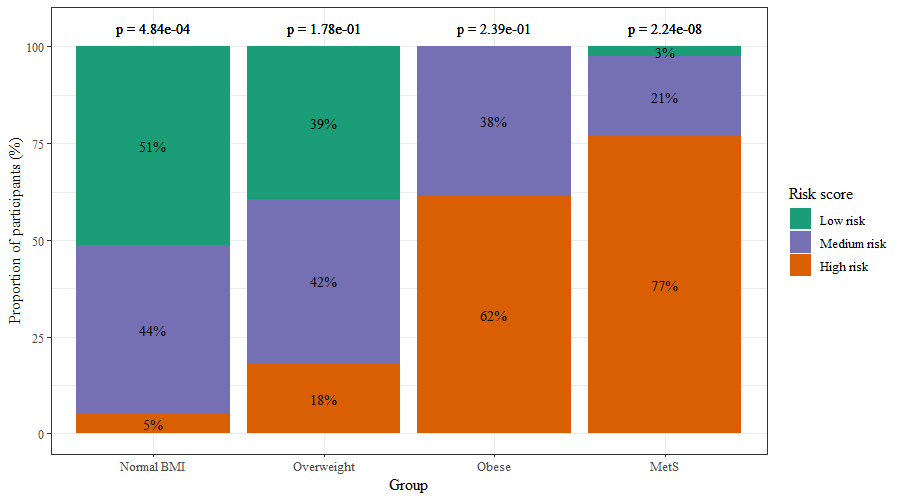


**Supplementary Figure 1. Risk of participants developing T2DM**. Disease risk was calculated from the AUSDRISK questionnaire (see details in **Methods - 2.6.3**). P-values are from chi squared analysis for homogeneity.


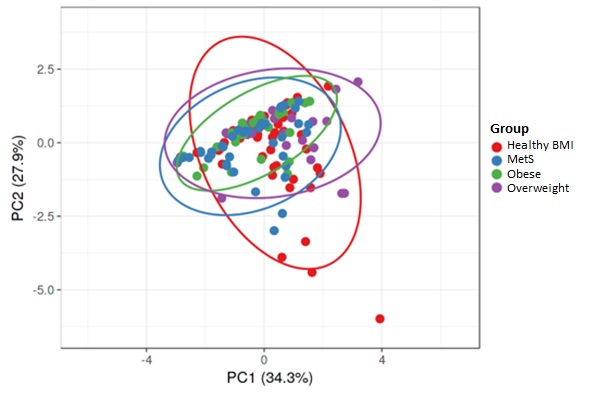


**Supplementary Figure 2.** Partial least squares discriminant analysis (PLS-DA) highlights the consistency of microbial composition, using phyla level data, between groups.

**Supplementary Figure 3. Sex differences in TMAO within total study population and experimental groups.** Differences in fasting circulating TMAO were investigated between male and female participants within the full study population (**A**; *n*=70 for Males; *n*=66 for Females), and within the experimental groups (**B**; *n*=17 for Healthy BMI Males; *n*=23 for Normal BMI Females; *n*=14 for Overweight Males; *n*=19 for Overweight Females; *n*=11 for Obese Males; *n*=15 for Obese Females; *n*=29 for MetS Males; *n*=9 for MetS Females). Data is expressed as means±SEM. A student t-test was used to detect differences between the two study populations (**A**), whilst a two-way ANOVA was used for the latter analyses (**B**). In particular, a Šidák’s multiple comparisons test was used to compare between male and female data within each experimental group, whilst a Tukey’s multiple comparisons test was used to compare the means of each experimental group within the sexes.

**
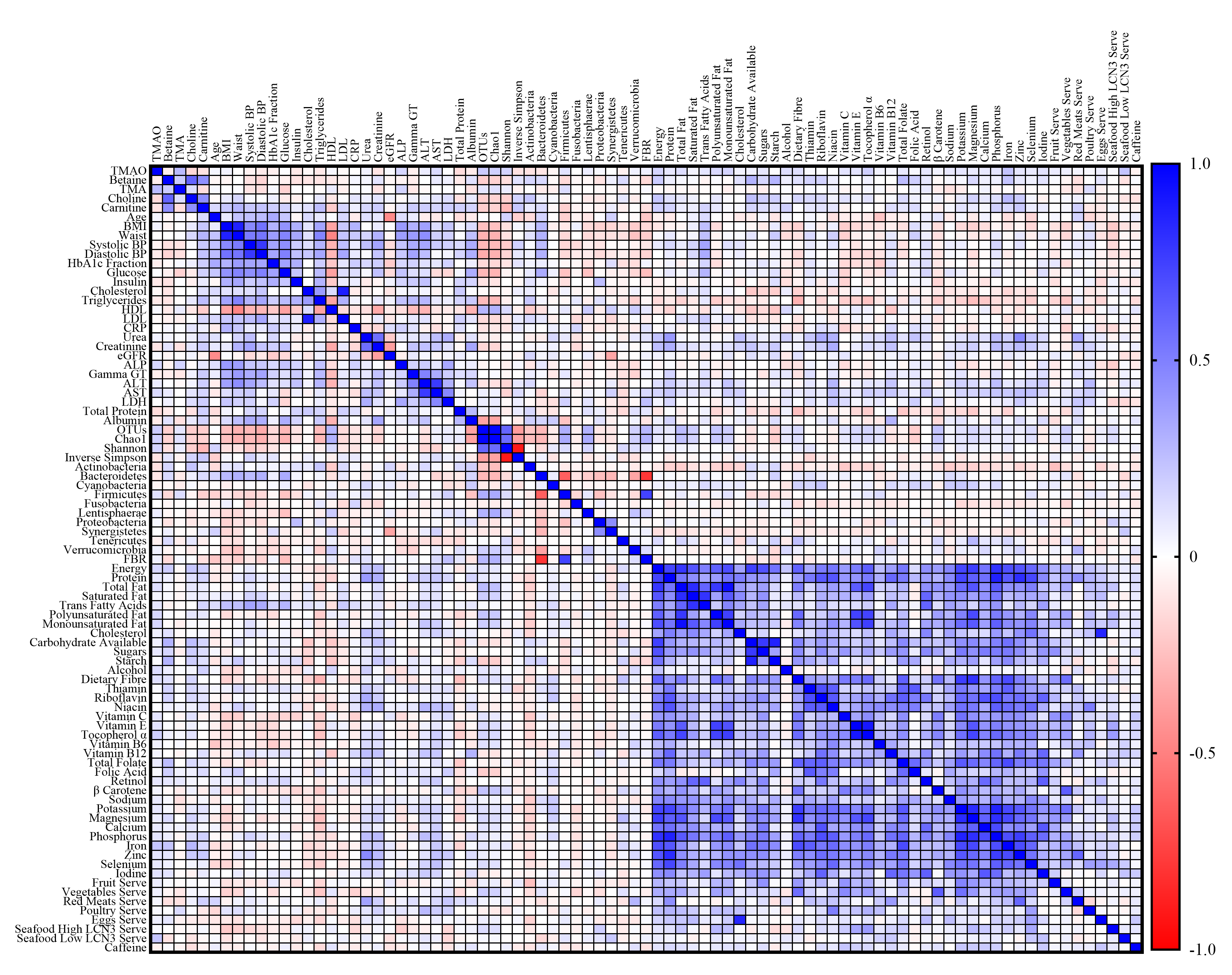
**

**Supplementary Figure 4. Correlation heatmap for all variables across study population.** A correlation matrix with a Pearson’s correlation coefficient (r) calculated for every pair of data sets in the study population is presented in the form of a heatmap. *n*=140.

| **Supplementary Table 1. HPLC Gradient Conditions** | |
| --- | --- |
| **Time (min)** | **% B** |
| 0.01 | 20 |
| 0.20 | 20 |
| 15.00 | 98 |
| 20.00 | 98 |
| 20.10 | 20 |
| 25.00 | 20 |

| **Supplementary Table 2. Compound- And Instrument-Specific Scheduled Multiple Reaction Monitoring Parameters In Positive Ionisation Mode.** Up to three transitions were chosen for each target compound. The first transition ion was used as the quantifier while other transitions were used as qualifiers. Interface potential for all compounds was set at 0.75 kV. | | | | | | | |
| --- | --- | --- | --- | --- | --- | --- | --- |
| **Compound** | **Retention Time (min)** | **Precursor m/z** | **Product m/z** | **Dwell Time (msec)** | **Q1 Pre Bias (V)** | **Collision Energy** | **Q3 Pre Bias (V)** |
| TMAO | 1.01 | 76.10 | 58.15 | 25 | -12.0 | -21.0 | -24.0 |
|  |  |  | 59.20 | 25 | -12.0 | -16.0 | -23.0 |
|  |  |  | 42.10 | 25 | -12.0 | -38.0 | -16.0 |
| TMAO-D9 | 1.02 | 85.00 | 66.25 | 25 | -14.0 | -21.0 | -12.0 |
|  |  |  | 68.20 | 25 | -13.0 | -16.0 | -12.0 |
|  |  |  | 46.20 | 25 | -28.0 | -39.0 | -18.0 |
| TMA | 0.99 | 60.40 | 44.15 | 25 | -12.0 | -22.6 | -17.0 |
|  |  |  | 45.15 | 25 | -12.0 | -17.1 | -18.0 |
|  |  |  | 29.90 | 25 | -16.0 | -42.1 | -26.0 |
| Carnitine | 0.98 | 162.20 | 103.1 | 25 | -26.0 | -15.2 | -18.0 |
|  |  |  | 28.90 | 25 | -28.0 | -47.2 | -14.0 |
|  |  |  | 58.10 | 25 | -17.0 | -37.4 | -25.0 |
| Choline | 0.98 | 104.11 | 60.15 | 25 | -22.0 | -18.6 | -20.0 |
|  |  |  | 58.15 | 25 | -23.0 | -30.5 | -20.0 |
|  |  |  | 45.15 | 25 | -10.0 | -21.8 | -18.0 |
| Betaine | 1.06 | 117.90 | 58.10 | 25 | -26.0 | -26.8 | -23.0 |
|  |  |  | 59.20 | 25 | -24.0 | -20.8 | -25.0 |
|  |  |  | 42.10 | 25 | -25.0 | -53.7 | -17.0 |
| AZT | 1.71 | 268.00 | 127.05 | 25 | -22.0 | -11.5 | -21.0 |
|  |  |  | 110.05 | 25 | -20.0 | -30.2 | -11.0 |
|  |  |  | 109.15 | 25 | -13.0 | -30.2 | -11.0 |

Dietary macro- and micronutrient intake was recorded from a three-day self-reported food diary which had been analysed using FoodWorks Software (Xyris, Brisbane, Australia) and daily averages calculated (**Supplementary Table 3**). Energy and macronutrient intake of protein, total fat and carbohydrates did not differ across the groups. Intake of fat macronutrients (trans fatty acids, poly- and monounsaturated fatty acids) together with cholesterol intake were unchanged between the groups. For fat macronutrients, intake of trans fatty acids was significantly higher in those with obesity when compared to participants with a healthy BMI (*p*<0.05). Total carbohydrates, sugar and starch intake, along with dietary fibre were unchanged across groups, Alcohol, vitamin and mineral intake were generally similar between the groups, though vitamin C intake was significantly lower in subjects with obesity (*p*<0.05). Intake of fruits, vegetable, red meat, and poultry did not differ, while consumption of eggs was lowest in those with MetS (*p*<0.05). Consumption of seafood with higher n-3 long chain fatty acids was also lowest in participants with MetS (*p*<0.05). Caffeine intake was lowest in those who were overweight (*p*<0.05).

| **Supplementary Table 3. Dietary Macro- and Micronutrients** | | | | | |
| --- | --- | --- | --- | --- | --- |
|  | **Healthy BMI** | **Overweight** | **Obese** | **MetS** | ***p*** |
| **Energy (kJ)** | 8374±365 | 8268±409 | 8462±504 | 8792±516 | NS |
| **Protein (g)** | 99.0±5.4 | 97.9±6.8 | 98.3±5.1 | 104.0±5.1 | NS |
| **Total fat (g)** | 79.2±4.8 | 80.2±43.1 | 84.5±7.3 | 81.4±5.7 | NS |
| **Saturated fat (g)** | 28.0±1.9 | 30.4±187 | 31.3±2.2 | 32.6±3.1 | NS |
| **Trans Fatty Acids (g)** | 1.0±0.1 | 1.2±0.1 | 1.4±0.1* | 1.4±0.1* | *, *p*<0.05 *vs*. Normal BMI |
| **Polyunsaturated fat (g)** | 14.0±1.4 | 12.2±0.9 | 13.2±1.5 | 11.1±0.8 | NS |
| **Monounsaturated fat (g)** | 30.5±2.0 | 31.0±1.8 | 33.1±3.3 | 31.0±2.3 | NS |
| **Cholesterol (mg)** | 379.7±45.5 | 335.6±29.1 | 424.4±50.3 | 314.7±25.6 | NS |
| **Total Carbohydrate (g)** | 191.0±10.5 | 179.3±10.6 | 190.3±14.2 | 213.3±19.6 | NS |
| **Sugars (g)** | 88.0±5.5 | 79.7±6.6 | 79.9±6.1 | 99.2±13.9 | NS |
| **Starch (g)** | 100.6±7.1 | 95.8±6.3 | 108.8±9.1 | 111.7±8.8 | NS |
| **Alcohol (g)** | 10.3±2.5 | 13.3±3.5 | 9.3±3.3 | 8.9±3.3 | NS |
| **Dietary fibre (g)** | 27.8±1.9 | 25.0±2.3 | 22.4±1.9 | 21.7±1.6 | NS |
| **Thiamine (mg)** | 1.6±0.2 | 1.5±0.2 | 1.5±0.1 | 2.0±0.2 | NS |
| **Riboflavin (mg)** | 2.2±0.2 | 2.0±0.2 | 2.±0.1 | 2.6±0.3 | NS |
| **Niacin (mg)** | 24.4±2.5 | 24.3±2.0 | 25.2±1.6 | 27.3±2.0 | NS |
| **Vitamin C (mg)** | 128.6±16.4 | 109.8±14.5 | 77.9±6.9* | 99.0±13.2 | *, *p*<0.05 *vs*. Normal BMI |
| **Vitamin E (mg)** | 13.7±1.3 | 11.7±1.1 | 12.7±2.0 | 12.7±1.4 | NS |
| **Tocopherol α (mg)** | 11.5±1.0 | 10.7±1.0 | 10.3±1.2 | 11.0±1.2 | NS |
| **Vitamin B6 by analysis (mg)** | 1.9±0.3 | 1.6±0.2 | 1.9±0.3 | 1.5±0.1 | NS |
| **Vitamin B12 (µg)** | 5.2±0.5 | 4.6±0.3 | 4.6±0.3 | 5.4±0.4 | NS |
| **Total folate (µg)** | 552.3±36.7 | 499.1±37.8 | 469.8±33.0 | 516.9±39.1 | NS |
| **Folic acid (µg)** | 132.8±16.2 | 151.6±21.4 | 138.5±15.8 | 192.3±26.2 | NS |
| **Retinol (µg)** | 303.2±25.6 | 303.1±28.3 | 339.5±33.0 | 324.5±28.5 | NS |
| **Beta carotene (µg)** | 4121±453 | 4033±1007 | 2934±600 | 3127±726 | NS |
| **Sodium (mg)** | 2288±148 | 2685±185 | 2571±154 | 2355±140 | NS |
| **Potassium (mg)** | 3442±187 | 3185±247 | 2876±193 | 3235±198 | NS |
| **Magnesium (mg)** | 408.±27 | 388±38 | 347±26 | 365±23 | NS |
| **Calcium (mg)** | 967±60 | 970±77 | 866±67 | 1013±75 | NS |
| **Phosphorus (mg)** | 1628±83 | 1580±103 | 1601±85 | 1658±89 | NS |
| **Iron (mg)** | 12.2±0.8 | 11.7±1.0 | 11.1±0.8 | 12.5±1.1 | NS |
| **Zinc (mg)** | 11.1±0.6 | 10.8±0.7 | 10.8±0.7 | 12.6±0.8 | NS |
| **Selenium (µg)** | 94.9±6.4 | 99.3±12.0 | 91.3±6.2 | 84.5±4.1 | NS |
| **Iodine (µg)** | 171.2±10.1 | 180.1±17.1 | 174.8±11.2 | 187.1±12.7 | NS |
| **Fruit (serve)** | 1.4±0.2 | 1.3±0.2 | 0.9±0.2 | 0.9±0.1 | NS |
| **Vegetables (serve)** | 4.0±0.3 | 3.9±0.5 | 3.6±0.5 | 3.4±0.3 | NS |
| **Red meats (serve)** | 0.6±0.3 | 0.6±0.1 | 0.5±0.1 | 1.1±0.2 | NS |
| **Poultry (serve)** | 0.4±0.1 | 0.6±0.1 | 0.7±0.1 | 0.6±0.1 | NS |
| **Eggs (serve)** | 0.4±0.1 | 0.3±0.0 | 0.4±0.1 | 0.2±0.0* | *, *p*<0.05 *vs*. Normal BMI |
| **Seafood (high L-C N3 serve)** | 0.2±0.1 | 0.2±0.0 | 0.1±0.0 | 0.1±0.1* | *, *p*<0.05 *vs*. Normal BMI |
| **Seafood (low L-C N3 serve)** | 0.2±0.1 | 0.1±0.0 | 0.2±0.1 | 0.1±0.0 | NS |
| **Caffeine (mg)** | 192.0±25.8 | 119.1±15.1* | 128.3±16.8 | 172.9±18.7 | *, *p*<0.05 *vs*. Normal BMI |
| Dietary intakes of micro- and macro-nutrients was determined for participants using the FoodWorks Software based off as 3-day food diary. Data are presented as mean±SEM and compared via a one-way ANOVA using an initial Tukey’s post hoc analysis for all comparisons as an exploratory tool to identify changes between groups. A subsequent one-way ANOVA was also used to compare all means against the Healthy BMI group in conjunction with a Dunnet’s post-hoc test to increase statistical sensitivity. Abbreviations: L-C N3, long-chain N-3 polyunsaturated fatty acids. P-values shown for different inter-group comparisons | | | | | |

| **Supplementary Table 4. Medications Taken by Participants.** | | | | |
| --- | --- | --- | --- | --- |
| Disease/Condition Grouping | Healthy BMI | Overweight | Obese | MetS |
| Infectious Disease |  |  |  |  |
| Antiviral | 1 | 0 | 0 | 0 |
| Antimalarials | 1 | 0 | 0 | 0 |
| Contraception/Menstrual health/Hormones |  |  |  |  |
| Cyproterone & ethinylestradiol (oral) | 2 | 0 | 0 | 1 |
| Implanon (progesterone) | 1 | 2 | 0 | 0 |
| Antineoplastics | 1 | 0 | 0 | 0 |
| Estradiol | 1 | 0 | 0 | 0 |
| Dydrogesterone & estradiol hemihydrate | 1 | 0 | 0 | 0 |
| Ethinyl estradiol & drospirenone | 0 | 1 | 0 | 0 |
| SERM | 1 | 0 | 0 | 0 |
| 5α reductase inhibitors | 0 | 1 | 0 | 0 |
| Testosterone | 0 | 0 | 0 | 1 |
| Hormone replacement therapy (for women) | 0 | 1 | 0 | 1 |
| Osteoporosis |  |  |  |  |
| Bisphosphonate | 1 | 0 | 0 | 0 |
| Asthma |  |  |  |  |
| LABA | 2 | 2 | 0 | 2 |
| SABA | 1 | 1 | 1 | 3 |
| Corticosteroids | 2 | 0 | 0 | 0 |
| Antidepressant/psychiatric/Neuro |  |  |  |  |
| SSRI | 3 | 0 | 0 | 0 |
| SNRI | 0 | 1 | 1 | 0 |
| Tricyclic | 1 | 1 | 0 | 0 |
| Tetracyclic | 0 | 1 | 0 | 0 |
| Benzodiazepine | 0 | 1 | 0 | 2 |
| Dopamine agonist | 0 | 1 | 0 | 2 |
| Anticholinergic | 0 | 1 | 0 | 0 |
| Anticonvulsants | 0 | 0 | 2 | 1 |
| MT_1_ and MT_2_ agonist | 1 | 0 | 0 | 0 |
| Hay fever |  |  |  |  |
| Antihistamines | 1 | 1 | 0 | 0 |
| Thyroid |  |  |  |  |
| Thyroid replacement | 4 | 1 | 1 | 0 |
| Gastrointestinal |  |  |  |  |
| Proton pump inhibitors | 1 | 2 | 2 | 3 |
| H_2_ blockers | 0 | 1 | 1 | 0 |
| DA_2_ blocker | 0 | 0 | 1 | 0 |
| Aminosalicylates | 0 | 0 | 0 | 2 |
| Pain medication/Immune modulators |  |  |  |  |
| Anti-histamines | 0 | 0 | 1 | 0 |
| COX-2 inhibitor | 1 | 1 | 0 | 1 |
| COX-1 & COX-2 inhibitor | 1 | 1 | 0 | 9 |
| Corticosteroids | 1 | 0 | 0 | 0 |
| JAK inhibitor | 1 | 0 | 0 | 0 |
| Paracetamol | 0 | 0 | 0 | 3 |
| Serotonin receptor agonists | 0 | 2 | 0 | 0 |
| Opioids | 0 | 1 | 0 | 4 |
| Lipid handling |  |  |  |  |
| Fibrate | 0 | 0 | 0 | 1 |
| Statin | 3 | 1 | 0 | 6 |
| NPC1L1 inhibitor | 0 | 0 | 0 | 2 |
| Blood pressure |  |  |  |  |
| β1-blocker | 2 | 0 | 0 | 3 |
| ACE inhibitor | 0 | 1 | 1 | 2 |
| ARB | 0 | 1 | 1 | 4 |
| Ca^2+^ channel inhibitor | 0 | 1 | 0 | 0 |
| Blood clotting |  |  |  |  |
| Factor Xa inhibitors | 2 | 1 | 0 | 0 |
| Antiplatelet | 0 | 0 | 0 | 1 |
| Heart failure |  |  |  |  |
| Digitalis glycosides | 1 | 0 | 0 | 0 |
| Metabolic Control |  |  |  |  |
| Anorectics | 0 | 1 | 0 | 0 |
| Xanthine oxidase inhibitors | 0 | 0 | 1 | 1 |
| Eye Medication |  |  |  |  |
| β-blocker | 1 | 0 | 0 | 0 |
| Abbreviations: ACE, angiotensin converting enzyme; ARB, Angiotensin receptor blockers; DA_2_, dopamine receptor D2; H_2_, histamine H2 receptor; JAK, janus kinase; LABA, long-acting β2 agonist; MT, Melatonin receptor agonist; NPC1L1, Niemann-Pick C1-Like 1 protein; SABA, short-acting β2 agonist; SERM, selective estrogen receptor modulator; SNRI, serotonin-norepinephrine reuptake inhibitor class; SSRI, selective serotonin reuptake inhibitor; TSH, thyrotropin; COX, cyclooxygenase. | | | | |
